# Critically appraised topic on adverse food reactions of companion animals (5): discrepancies between ingredients and labeling in commercial pet foods

**DOI:** 10.1186/s12917-018-1346-y

**Published:** 2018-01-22

**Authors:** Thierry Olivry, Ralf S. Mueller

**Affiliations:** 1 Department of Clinical Sciences, College of Veterinary Medicine, North Carolina State University, 1060 William Moore Drive, Raleigh, NC 27607 USA; 2 Medizinische Kleintierklinik, Centre for Clinical Veterinary Medicine, Ludwig Maximilian University, Veterinärstrasse 13, 80539 Munich, Germany

**Keywords:** Canine, Cat, Contamination, Diet, Dog, Feline, Food allergy, Labeling, Pet foods

## Abstract

**Background:**

Elimination dietary trials for the diagnosis of adverse food reactions (food allergies) in dogs and cats are often conducted with commercial pet foods while relying on their label to select those not containing previously-eaten ingredients. There are concerns that industrial pet foods might contain unlisted food sources that could negate the usefulness of performing food trials. Furthermore, unidentified ingredients might cause clinical reactions in patients hypersensitive to such items.

**Results:**

We searched two article databases on July 7, 2017 and January 12, 2018 for relevant articles, and we screened abstracts from the leading international veterinary dermatology congresses for suitable material. Additional citations were found in the selected papers. In all, we extracted data from 17 articles and one abstract. The studies varied both in the number of pet foods tested (median: 15; range: 1 to 210) and that of ingredients specifically evaluated (median: 4; range: 1 to 11). Studies most often employed either PCR to detect DNA or ELISA to identify proteins from one or more vegetal or animal species; two studies used mass spectrometry to increase the number of detectable proteins. The various methods found ingredients that were not on the label in 0 to 83% (median: 45%) of tested diets; this percentage varied between 33 and 83% in pet foods with “novel/limited” ingredients proposed for elimination diets. Similarly, ingredients were found to be missing from the label in 0 to 38% (median: 1%) of tested foods. Finally, six studies evaluated, among others, several hydrolysate-containing pet foods: mislabeling with unlabeled or missing ingredients was found only in one diet.

**Conclusions:**

The mislabeling of pet foods appears rather common, even in those with “novel” or “limited” ingredients proposed for elimination diets. Unexpected added ingredients are more frequently detected than those missing from the label. There is insufficient information to determine if the presence of a contaminating component will lead to a clinical reaction in a patient allergic to it, as challenges with the mislabeled foods were not performed in dogs or cats allergic to such ingredients. The testing of hydrolysate-containing pet foods found only one instance of possible mislabeling.

**Electronic supplementary material:**

The online version of this article (10.1186/s12917-018-1346-y) contains supplementary material, which is available to authorized users.

## Background

To diagnose an adverse food reaction (i.e. food allergy) in a canine or feline patient, a lengthy period of dietary restriction is followed by sequential food challenges to identify the culprit food allergen(s). At this time, the restriction phase is most commonly performed by feeding commercially-available pet foods while relying on the ingredients written on the label to select an elimination diet not containing previously eaten food items. Likewise, after the diagnosis of adverse food reaction is made, the patient is maintained on a diet expected not to contain the offending allergens. At this stage again, veterinarians and pet owners are relying on the proper labeling of commercial pet foods to select a diet not containing the offending allergens. There are concerns that commercial foods may be contaminated with unexpected ingredients, either at the stage of ingredient selection or during their manufacturing. The rate of pet food with incorrect labeling, either with ingredients not on, or missing from, the label is not widely known.

## Clinical scenario

Your patient is a 3-year-old male castrated German shepherd dog that you had diagnosed four years ago with food-induced atopic dermatitis. A previous dietary trial had established that this dog’s pruritus and skin lesions had completely resolved following an elimination diet with a hydrolysate-containing pet food. Signs had relapsed in the day following an oral challenge with chicken, an ingredient present in its first adult diet. During the past two years, this dog had been eating a fish-and-rice dog food, and its atopic dermatitis had remained in complete clinical remission. Due to a problem with the supply of his usual pet food, the owner recently changed it to another fish-and-rice over-the-counter diet from a different manufacturer. Within one day of starting the new diet, this dog began having a severely pruritic erythematous patchy and papular eruption on the abdomen and axillae. The owner does not understand why her dog is having a flare of atopic dermatitis, as the ingredients listed on the diet’s label are similar to those eaten previously. You suspect that this new diet might include some chicken and you wonder how commonly pet food ingredient mislabeling occurs.

## Structured question

*Do commercial pet foods commonly contain ingredients not listed on their labels?*

## Search strategy

We queried the Web of Science Core Collection and CAB Abstract databases on two occasions, July 7, 2017 and January 12, 2018 with the following search string: (dog or dogs or canine or cat or cats or feline or pet or pets) and (food* or diet*) and commercial and (contamination or ingredient* or antigen* or allergen*). Altogether, the search was limited to the years 2000–2017, and there was no limit of publication language. The bibliography of each selected article was then screened for additional papers that could be of interest. We also looked for relevant information in the published abstracts from North American, European and world congresses of veterinary dermatology. Finally, additional database searches were done with the first author’s names of identified abstracts of interest to determine if full-length articles later reported the same material.

## Identified evidence

Our widest search identified 145 and 151 citations in the Web of Science and CAB abstracts, respectively. Among these, we found only three relevant articles [1–3], two of which were included in both databases. The search of the published congress abstracts yielded four additional summaries [4–7], and a further query of databases with the abstracts’ lead author’s name resulted in three articles missed by our original search [8–10]. Finally, 12 additional articles were identified from screening the bibliography of previously-selected papers [11–22] Altogether, we reviewed the information included in 18 articles and one abstract [7]. One paper was later discarded, as it only dealt with treats and supplements and not pet foods [17]. In all, we retained 18 reports of pertinent information.

## Evaluation of evidence

The studies had been conducted with diets purchased in seven countries: the USA (five studies) [2, 3, 8, 12, 18], Spain (five–all from the same research group) [14–16, 19, 20], Italy (two) [1, 11], Taiwan (two) [13, 22], France (two) [7, 10], and the United Kingdom [21] and Austria [9] with one each (Additional file 1: Table S1). The tested diets had been purchased in the general distribution or from veterinarians, and they consisted of either dry and wet foods or treats for everyday use. In six instances, the tested pet foods had been proposed as an aid for the diagnosis or management of adverse food reactions, as they were labelled to contain “limited” or “novel” [1, 8, 9, 18] or hydrolyzed ingredients [1, 7, 9, 10, 18] (Additional file 1: Table S1). Regrettably, only six articles specifically reported the brand of the tested diets [7–10, 18, 21].

The studies varied both in the number of pet foods tested (median: 16; range: 1 to 210) and that of ingredients specifically evaluated (median: 4; range: 1 to 11). All but three studies employed PCR techniques to amplify the DNA from a single (e.g. beef, chicken, wheat…) or a group of species (e.g. avian, fish, poultry, leporids…). Two reports from the same group mentioned ELISAs being used to test for the presence of one or more protein source [8, 18]. Finally, it is only in the two last studies that one of the methods used (i.e. mass spectrometry) might, at least in theory, permit the detection of a potentially infinite number of contaminating proteins [7, 10].

**Fig. 1:**
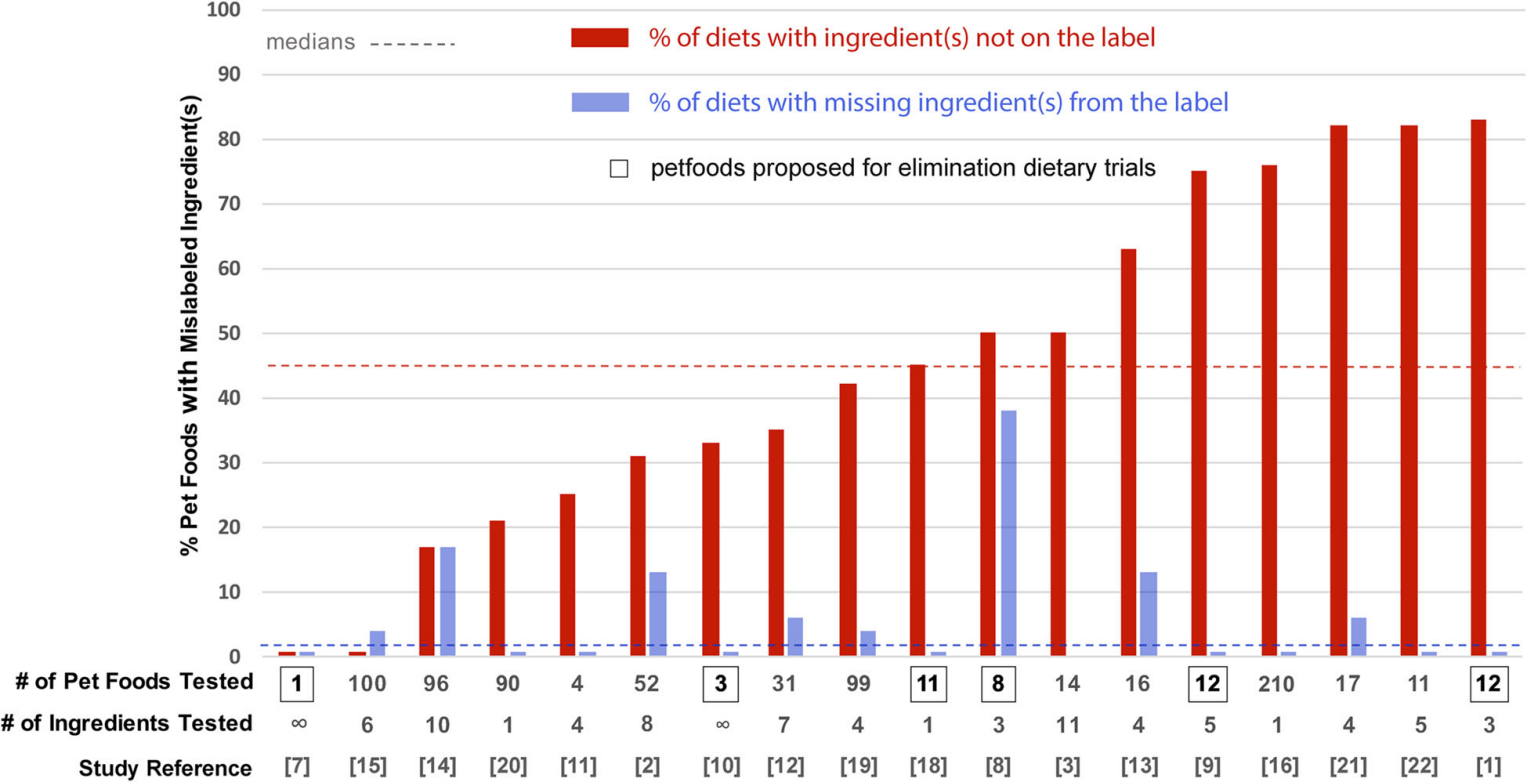
Proportions of pet foods with added mislabeled ingredients and those with missing ingredients from the label. The percentage of diets with added mislabeled ingredients are in red, those with missing ingredients from the label are in blue. Dotted lines represent the respective medians. Bolded and squared numbers represent studies with foods with “novel”, “limited” or hydrolyzed ingredients that had been selected because of their proposed used for elimination diets. The infinite symbols represent the studies using mass spectrometry that, theoretically, should allow for the detection of an infinite number of contaminating proteins

We analyzed the study results both for data suggesting the presence of unlabeled potentially allergenic ingredients, but also for evidence demonstrating the absence of components written on the label (Additional file 1: Table S1). Altogether, the percentage of tested pet foods with mislabeled ingredients varied from 0 to 83% (median: 45%; Additional file 1: Table S1; Fig. 1). Even in studies testing pet foods with “novel/limited” ingredients proposed for elimination (i.e. restrictive) diets, the percentage of mislabeling varied from 33 to 83% of evaluated foods [1, 8–10, 18]. Interestingly, except for one instance [10], the pet foods with hydrolyzed proteins were not found to contain protein sources from an unexpected species [1, 7, 9, 18]. In the study with potential mislabeling of a hydrolysate-containing diet, the authors considered the possibility of cross-contamination, but they also discussed the hypothesis that a previously unknown potato protein homologous to the detected rice protein might have been missing from the database used to match the sequences identified by mass spectrometry [10].

Overall, the identity of the mislabeled ingredient(s) varied depending upon the method of testing, which had been set up to detect one or more protein or DNA source, and details are provided in Additional file 1 : Table S1.

In all but one article [3], the methods used (ELISA or PCR) permitted an estimation of the rate of ingredients missing from the label. Such missing ingredient(s) were found in 8/16 studies (50%; Additional file 1: Table S1) and the rate of mislabeled diets varied between 0 and 38% (median: 1%; Additional file 1: Table S1), a percentage negligible compared to that of ingredients not present on the label. Missing ingredients were not detected in the tested pet foods that contain hydrolysates.

## Limitations

This review of the existing evidence on pet food mislabeling highlighted several limitations that should be taken into consideration before translating these results to clinical practice. Firstly, it is very likely that the rate of pet food mislabeling, either due to unlabeled or missing food ingredients, is higher than that reported herein. This underestimation stems from the methods used that, except for the last studies that used mass spectrometry, only looked for the presence or absence of a small number of specific ingredients. Secondly, these reports usually would not be able to determine if the mislabeling was due to the cross-contamination of the food supply or manufacturing lines or to a deliberate misleading intent (i.e. a deliberate fraud). Finally—and most importantly—the presence of an ingredient not included in a pet food’s label does not imply that a patient allergic to this particular food source would have a clinical reaction to this contaminant. Indeed, an individual’s clinical reactivity depends upon a combination of factors including the type of allergen(s) recognized by the patient’s immune system, the amount of mislabeled allergen(s) present in the pet food and the degree of hypersensitivity of the patient itself.

## Conclusion and implication for practitioners

Our review of the literature suggests that the mislabeling of pet foods is rather common, even in those with “novel” or “limited” ingredients proposed for elimination diets. Unexpected added ingredients are more frequently detected than those missing from the label. There is insufficient information to determine if the presence of a contaminating ingredient will lead to a clinical reaction in a patient allergic to it, as challenges with the mislabeled foods were not performed in allergic dogs or cats allergic to such ingredients. The testing of hydrolysate-containing pet foods found only one instance with possible mislabeling.

## Additional file

Additional file 1: Table S1.
Studies summary. (PDF 26 kb)

## Abbreviations

CAT: Critically appraised topic
