# Supplementary material for: Critically appraised topic on adverse food reactions of companion animals (5): discrepancies between ingredients and labeling in commercial pet foods
Source: BMC Vet Res. 2018 Jan 22;14:24. doi: 10.1186/s12917-018-1346-y (PMC5778722; doi:10.1186/s12917-018-1346-y)
Supplement: Additional file 1: Table S1. — Studies summary. (PDF 26 kb) [file 12917_2018_1346_MOESM1_ESM.pdf]

Supplementary Table 1: studies summary

Author	Year	Ref #	Country	Method	# Food tested	Tested source of allergens	# (%) of diets with detected ingredient(s) not on the label	Identity of the source of allergen(s) not on the label *	# (%) of diets with ingredient(s) missing from the label	Identity of the source of allergen(s) missing from the label	Foods tested
Dalmasso	2004	[11]	Italy	PCR	4	fish, pork, poultry, ruminants	1 (25%)	poultry	0 (0%)	-	4 pet foods (brands not specified)
Myers	2004	[12]	USA	PCR	31	beef, cat, dog, horse, lamb, pork, poultry	11 (35%)	poultry > beef	2 (6%)	lamb, poultry	31 dog foods (brands not specified)
Wang	2004	[13]	Taiwan	PCR	16	beef, chicken, goat, pork	10 (63%)	not clearly specified	2 (13%)	chicken	8 cat and 8 dog foods (brands not specified)
Raditic	2011	[8]	USA	ELISA	8	beef, poultry, soy	4 (50%)	soy > beef	3 (38%)	chicken	8 dog foods: 4 "test" and 4 "control" diets; the test diets were labeled to contain venison but not beef or soy (brands mentioned)
Pegels	2013	[14]	Spain	PCR	96	beef, chicken, fish, hare, lamb, leporids, pork, rabbit, turkey, venison	16 (17%)	chicken	16 (17%)	rabbit > venison	96 cat and dog foods with a rabbit protein ingredient on the label (brands not specified)
Pegels	2013	[15]	Spain	PCR	100	beef, chicken, fish, pork, poultry, turkey	0 (0%)	-	4 (4%)	fish oil	100 cat, dog and fish foods wifh a fish protein ingredient on the label (brands not specified)
Ricci	2013	[1]	Italy	MBFI<br>PCR	12	avian, fish, mammalian DNA (PCR) or bones (MBFI)	10 (83%)	poultry > fish > mammals	0 (0%)	-	12 dog foods used for elimination diets: 11 with "novel" protein source and 1 with hydrolyzed proteins (brands not specified)
Pegels	2014	[16]	Spain	PCR	210	avian (multiple species)	66/87 (76%)	avian (species not characterized)	0/123 (0%)	-	210 cat and dog foods: 123 with an avian protein ingredient on the label and 87 without such ingredient on the label (brands not specified)
Pegels	2015	[19]	Spain	PCR	99	barley, oats, rye, wheat	30/72 (42%)	barley > oats >wheat > rye	1/27 (4%)	oats	99 cat and dog foods: 27 with a barley, oats, rye or wheat protein ingredient on the label and 72 without such protein ingredients on the label (brands not specified)
Willis-Mann	2014	[18]	USA	ELISA	11	soy	5 (45%)	soy	0 (0%)	-	11 dog foods: 4 labeled as "no soy" and 7 used for elimination diets; 3 of the latter contained hydrolyzed proteins (brands mentioned)
Maine	2015	[21]	United Kingdom	PCR	17	beef, chicken, horse, pork	14 (82%)	chicken > pork	1 (6%)	chicken	17 cat and dog wet foods (brands mentioned)
Pegels	2015	[20]	Spain	PCR	90	horse	12/57 (21%)	horse	0/33 (0%)	-	90 cat and dog foods: 33 with an horse protein ingredient on the label and 57 without such ingredient on the label (brands not specified)
Okuma	2015	[2]	USA	PCR	52	beef, chicken, goat, goose, horse, lamb, pork, turkey	16 (31%)	pork > turkey > chicken, beef > lamb	7 (13%)	beef > turkey > lamb, pork	52 cat and dog foods (34) and treats (18) (brands not specified)
Hsieh	2016	[22]	Taiwan	ELISA<br>PCR	11	beef, chicken, goat, lamb, pork	9 (82%)	pork > lamb > chicken	0 (0%)	-	11 dog foods, canned (brands not specified)
Kanakubo	2017	[3]	USA	PCR	14	beef, cat, dog, goat, horse, lamb, mouse, pork, rabbit, rat, venison	7 (50%)	beef > lamb > pork	NT	NT	14 cat and dog foods marketed as vegetarian or vegan (brands not specified)
Horvath	2017	[9]	Austria	PCR	12	beef, chicken, lamb, pork, turkey	9 (75%)	beef > lamb, pork	0 (0%)	-	12 dog foods used for elimination diets including 2 containing hydrolyzed proteins (brands mentioned)
Lesponne	2017	[7]	France	total DNA<br>MS	1	∞	0 (0%)	-	0 (0%)	-	1 hydrolyzed dog food used for elimination diet (brand mentioned)
Roitel	2017	[10]	France	IB<br>MS	3	∞	1 (33%)	rice	0 (0%)	-	3 hydrolyzed dog foods used for elimination diets (brands mentioned)

Abbreviations: ELISA: enzymed-liked immunosorbent assay; IB: immunoblotting, MBFI: microscopic bone fragment identification; MS: mass spectrometry; NT: not tested; PCR: polymerase chain reaction

∞: potential and theoretical detection of an infinite number of proteins *: a ">" denotes the order of frequency of detection, a comma implies an equivalent rate of detection
